# Efficacy of Spa Therapy, Mud-Pack Therapy, Balneotherapy, and Mud-Bath Therapy in the Management of Knee Osteoarthritis. A Systematic Review

**DOI:** 10.1155/2018/1042576

**Published:** 2018-06-25

**Authors:** Antonio Fraioli, Gioacchino Mennuni, Mario Fontana, Silvia Nocchi, Fulvia Ceccarelli, Carlo Perricone, Angelo Serio

**Affiliations:** ^1^UOC Medicina Interna, Terapia Medica e Medicina Termale, Scuola di Specializzazione in Medicina Termale, Dipartimento di Medicina Interna e Specialità mediche, Sapienza Università di Roma, Azienda Ospedaliera Universitaria Policlinico Umberto I, Roma, Italy; ^2^UOC Reumatologia, Dipartimento di Medicina Interna e Specialità mediche, Sapienza Università di Roma, Azienda Ospedaliera Universitaria Policlinico Umberto I, Roma, Italy; ^3^Università Campus Bio-Medico di Roma, Roma, Italy

## Abstract

**Background:**

Osteoarthritis (OA) is the most common musculoskeletal disease in the world. OA is the result of an inflammatory and degenerative process affecting the entire joint. Osteoarthritis, especially involving the knee, has a relevant socioeconomic impact in terms of drugs, hospital admissions, work absences, and temporary or permanent invalidity. Therapy of knee osteoarthritis is based on pharmacological and nonpharmacological measures.

**Methods:**

We conducted a systematic review of the studies published between 2002 and 2017 on spa therapy, mud-pack therapy, balneotherapy, and mud-bath therapy in the treatment of knee osteoarthritis in order to investigate the evidence of the efficacy of such treatment on pain, functional limitation, drug use, and quality of life. Overall, 35 studies were examined among which 12 were selected and included in the review if they are trial comparative. We have been able to illustrate the main results obtained in the individual studies and to elaborate these results in order to allow as much a unitary presentation as possible and hence an overall judgment.

**Results:**

Because the studies we reviewed differed markedly from one another in terms of the methods used, we were unable to conduct a quantitative analysis (meta-analysis) of pooled data from the 12 studies. For the purposes of the present review, we reevaluated the results of the different studies using the same statistical method, Student's* t*-test, which is used to compare the means of two frequency distributions. Among all the studies, the most relevant indexes used to measure effectiveness of spa therapy were improved including VAS, Lequesne, and WOMAC Score.

**Conclusions:**

The mud-pack therapy, balneotherapy, mud-bath therapy, and spa therapy have proved to be effective in the treatment and in the secondary prevention of knee osteoarthritis, by reducing pain, nonsteroidal anti-inflammatory drug consumption, and functional limitation and improving quality of life of affected patients.

## 1. Introduction

Osteoarthritis (OA) is the most common musculoskeletal disease in the world, especially in the elderly; nonetheless it should not be considered exclusively an aging disease. It affects approximately 10% of people over 60 years old with a significant impact on the quality of life of patients who are limited in carrying out normal daily activities and on the healthcare systems [1–3].

In Italy, rheumatic and musculoskeletal diseases (RMDs) are the second most common chronic condition in the population, and among them OA is the most frequent representing the main cause of people's disability [4]. The prevalence of OA varies according to the definition of OA, the specific joint(s) under study, and the characteristics of the study population [3].

According to ISTAT data, osteoarthritis affects 15.9% of the population; the prevalence is age-increasing and sex-dependent, being higher in women [5]. Symptomatic OA is generally defined by the presence of pain, aching, or stiffness in a joint with radiographic. On the basis of a survey conducted in northwest Italy, 27% of the general population (31,2% women and 22,1% men) were affected by joint pain, defined by any pain lasting more than four weeks [6]. OA was defined as an articular disease resulting from loss of cartilage integrity in association with modifications of the adjoining bone tissue, due to an imbalance between catabolic phenomena and chondrocytic repair phenomena [7]. Osteoarthritis is low-grade inflammatory disease of synovial joints and is now defined as “a disorder involving movable joints characterized by cell stress and extracellular matrix degradation initiated by micro- and macro-injury that activates maladaptive repair responses including pro-inflammatory pathways of innate immunity. The disease manifests first as a molecular derangement (abnormal joint tissue metabolism) followed by anatomic, and/or physiologic derangements (characterized by cartilage degradation, bone remodeling, osteophyte formation, joint inflammation and loss of normal joint function), that can culminate in illness.” Inflammatory flares may be associated with swelling, redness, and pain [8, 9]. Indeed, synovitis, which is secondary to metabolic, biochemical, and mechanical alterations, is of importance in its pathogenesis. For the purposes of classification, it should be specified whether the knee osteoarthritis is of unknown origin (idiopathic, primary) or is related to a known medical condition or event (secondary). Clinical criteria for the classification of idiopathic OA of the knee were developed through a multicenter study group [10]. Regarding the prevalence of symptomatic knee OA in Italy, it ranges from 5.39% to 29.8% with an age-increasing incidence from 27% of subjects under the age of 70 to 44% of patients over 80 years of age. Moreover, women are more affected than men (11% versus 7%) [6, 11, 12]. OA, particularly knee osteoarthritis, has a high socioeconomic impact in terms of drug spending, hospital admissions, work productivity, and temporary or permanent incapacity [13, 14]. Treatment of OA is based on pharmacological and nonpharmacological measures [15]. The latter include the use of spa therapy (in a broad sense, balneotherapy and/or mud-pack therapy) integrated or alternated with other therapeutic prescriptions. Also, spa therapy (multiple interventions in spa resorts and in particular of mud-bath therapy) can be considered a cost-saving measure in the management of knee OA [16, 17]. Mud is a heated slurry, which is the result of the combination of solid material (mainly clay) and mineral water, used for external application after an adequate maturation period, at a temperature between 45°C and 50°C for the duration of 20-30 minutes. The baths consist of diving a patient in a bathtub with thermal (with a temperature of 36–38°C) and mineral (with high mineral content) water for 20 min. The spa therapy comprises a broad spectrum of therapeutic modalities including mud-pack therapy, balneotherapy, mud-bath therapy, hot showers, massage, and supervised water exercises in spa resorts, adding other benefits as a pleasant climate, relaxing natural scenery, and clean air [18]. The objective of this review is to summarize the currently available information on clinical effects and briefly discuss the possible mechanisms of action of spa therapy in knee OA.

## 2. Methods

**Figure 1:**
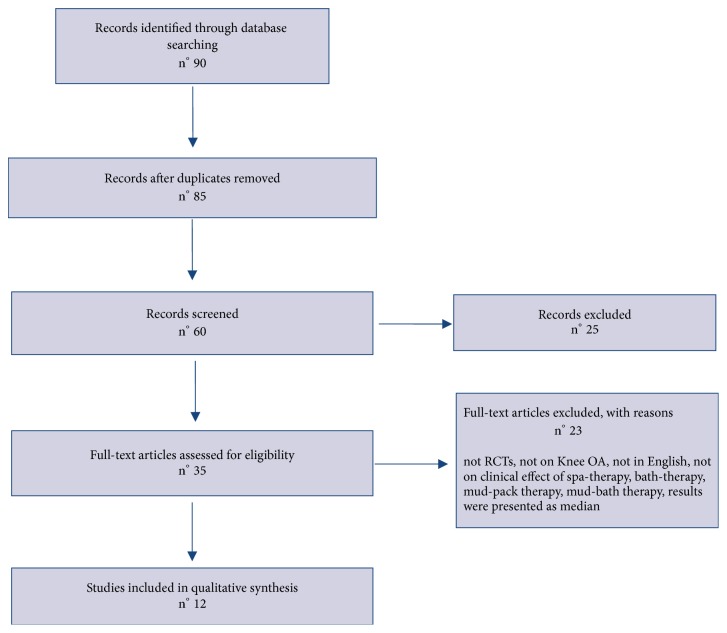
Study Flow Diagram.

We conducted a systematic review of the literature on spa therapy in the treatment of knee osteoarthritis in order to investigate the evidence of the efficacy of this treatment. We searched MEDLINE via PubMed for articles published between 2002 and 2017 using the terms “osteoarthritis”, “knee osteoarthritis”, “SPA therapy”, “mud-bath therapy”, and “randomized controlled clinical trials”. Studies were included in this review if in accordance with the eligibility criteria: clinical trials with patients conforming to the American College of Rheumatology (ACR) criteria relating to knee OA [10]; randomized controlled clinical trials (RCTs); clinical trials whose the main objective was the effectiveness of spa therapy [10]. The studies that were excluded from the review were those that analyzed the effects of spa therapy in different joints other than the knee, reviews, and those that are not in English and not on clinical effect of spa therapy, bath therapy, mud-pack therapy, and mud-bath therapy; moreover the studies in which data are expressed as median, with percentage but without absolute values or only with a graphic representation, were excluded. Overall, 35 studies were examined among which 12 were selected and included in the review if they were trial comparative. The Study Flow Diagram according to the Prisma Statement is reported in Figure 1 [19].

Each report was reviewed to identify the criteria used for study enrolment and for assignment to experimental versus control groups, sample size, type and characteristics of treatment, features of mineral water, control intervention, assessment point, endpoints, outcome measures, and tests used for statistical analysis of the results. Instead, data from each study were critically analyzed and the main findings compared with those of the other studies. For the quality assessment of the studies that were included in the review after the preliminary selection we considered the scientific value of the international journals that published these researches, the number of patients included in the studies, the methods used to study the patients, and the possibility of exclusion of more frequent studies bias. Moreover, we cannot exclude the existence of publication bias that can occur for any scientific research especially if related to topics concerning therapeutic methods.

Of 12 studies included in the review, 5 were carried out in Italy: 2 in* Chianciano SPA (Siena)* [20, 21], 2 in* Rapolano SPA (Siena)* [22, 23], and 1 in* Levico SPA (Trento)* [24]. The remaining 7 studies were carried out in other European or non-European countries, namely, 1 in France [25], 3 in Hungary [26–28], 2 in Turkey [29, 30], and 1 in Israel [31]. Overall studied subjects were 1044 of which 582 in the experimental groups and 462 in the control groups (by excluding patients treated only by physical therapy and those not completing the study, the total number of subjects evaluated in this review is 553), although no control group was included in two researches [23, 28]. The total number of patients according to the American College of Rheumatology (ACR) [10] criteria included in each study varies considerably from 382 to 20. Number of patients included in the experimental and control groups and treatment characteristics is shown in Tables 1, 2, 3, and 4. Four types of spa therapy were employed for patients placed in the experimental groups: mud-bath therapy in 5 studies (Table 1) [20–24], bath therapy in 5 studies (Table 2) [26–29, 31], mud-pack therapy in 1 study (Table 3) [30], and spa therapy (massages, showers, mud, and pool sessions) alone in 1 study (Table 4) [25] while patients in the control groups were allowed to continue their ongoing therapy. Two studies were double blinded using tap water in the control groups [26, 27].

Even though the methods used to evaluate the results of the therapy of the experimental groups may vary across studies, they are generally based on quantitative evaluations. In all studies the end points were evaluated at the beginning and at the end of the therapy but in some studies additional evaluation schedules were observed. Statistical analysis was performed using common tests (Student's* t*-test, Pearson's *χ*^2^ test, Wilcoxon's test, Z value, Mann–Whitney* U* test, and Friedman's test). Instead, data from each study were critically analyzed and the main findings compared with those of the other studies. For the purposes of the present review, we reevaluated the results of the different studies using the same statistical method, Student's* t*-test, which is used to compare the means of two frequency distributions. Assessment of pain was performed in all studies through Visual Analogue Scale (VAS) [32]. Assessment of OA was performed by WOMAC (Western Ontario and McMaster Universities Osteoarthritis Index) [33] in 7 studies and by the Lequesne's Index [34] in 6 studies.

The research carried out in* Chianciano SPA (Siena)* tested the effects of three mud-bath therapy cycles once a day for 12 days with sulphate-bicarbonate-calcium-magnesium mineral water over 1 year's time in patients with knee OA treated with analgesics and nonsteroidal anti-inflammatory drug (NSAID's). The control group was only required to continue the ongoing therapy. The patients who were already being treated with anti-inflammatory agents and analgesics were divided into two groups: the first group also underwent spa therapy, and the second did not [20]. At the end of the trial period, all patients were evaluated with VAS, Lequesne's Index, and the physical examination of each knee to assess the persistence of pain at palpation and during flexion-extension movements [32, 34].

In the second study performed in* Rapolano SPA (Siena)*, a 14-day mud-bath therapy cycle with bicarbonate-sulfate mineral water was planned in the experimental group, while ongoing therapy was allowed in experimental and control groups. Patients were assessed at baseline time, after 2 weeks and after 3, 6, and 9 months after the beginning of the treatment [22]. VAS, WOMAC, and Lequesne's Index were used and also the Arthritis Impact Measurement Scales (AIMS) [32–35].

Another study at* Rapolano SPA (Siena)* evaluated patients with knee OA treated with a cycle of a mud-bath therapy with sulphurous-calcium-bicarbonate mineral water. Ongoing therapy was allowed. No control group was considered in the study [23]. VAS, Lequesne's Index, and leptin and adiponectin plasma levels were assessed at baseline and after 2 weeks, upon completion of the mud-bath therapy [32, 34].

Another study based in* Chianciano SPA (Siena)* evaluated 12 mud-bath therapy with sulphate-bicarbonate-calcium-magnesium mineral water applications provided within two weeks. Ongoing therapy was allowed in experimental and control group [21]. Patients were assessed at basal time and at the end of spa treatment period with VAS, WOMAC and serum levels of adiponectin, resistin, and visfatin [32, 33].

The study at* Levico SPA (Trento)* evaluated patients with knee OA and considered two experimental groups, one of which was treated with cycle mud-bath therapy with arsenical-ferruginous mineral water (3 weeks) and the other with short wave therapy for the same period. Both the experimental group and the control group patients were allowed to continue the ongoing therapy [24]. The results were evaluated at baseline, at the end of the treatment period, and 12 weeks later by VAS, Lequesne's Index, and AIMS [32, 34, 35].

The study performed in* Cserkeszölö Spa (Hungary)* provided for the patients of the experimental group a 15-day bath therapy cycle with sodium bicarbonate mineral water and for the control group a cycle of similar duration of baths with tap water (placebo treatment) [26]. Results were evaluated at baseline, at the end of the spa therapy, and 3 months later with VAS and symptom scores [32].

The study in* Chamei Yoav SPA (Israel)* evaluated the effects of only bath therapy with salso-sulphate-bicarbonate-calcium mineral water applied once a week for 6 consecutive weeks. Ongoing therapy was allowed in experimental and control groups. Evaluation was done at baseline, at weeks 4 and 6 and 4 weeks following completion of treatment (week 10) [31]. VAS, WOMAC, and Lequesne's Index were used in this study [32–34].

The research conducted in* Alaçati Baths (Turkey)* assessed if patients undergoing bath therapy consisting of two baths with sodium-chloride-sulphate-calcium-magnesium mineral water daily (morning and afternoon) for 10 consecutive days were able to discontinue ongoing therapy. Control group subjects continued ongoing pharmacological treatment [29]. The results were evaluated at baseline, at the end of balneotherapy, at 2 weeks, and during follow-up period at 12 and 24 weeks later by VAS and Lequesne's Index [32, 34].

In the study from SPA Hévíz* (Hungary)*, baths with sulphurous-carbonated-calcium-magnesium and very light radon of Lake Hévíz water were provided for 30 minutes, 5 times a week for 3 weeks in the experimental group, while the control group patients performed the same number with the same duration of baths with tap water (placebo treatment). Both the experimental group and the control group patients were allowed to continue the ongoing therapy [27]. The research evaluated the outcome of bath therapy at baseline, at the end of the treatment and after 15 weeks by VAS, Womac, angle of knee flexion, joint circumference, stair-climb time, and questionnaire of general health-related quality of life [32, 33, 36].

The study at* Bank SPA (Hungary)* evaluated patients with osteoarthritis of the knee underwent a 15-day course of balneotherapy with mineral water contains sodium bicarbonate, fluoride, and metaboric acid. The patients were allowed to take NSAID's or analgesic drug of their choice. No control group was considered in the study [28]. The results were evaluated before the start of balneotherapy and at least 2 weeks and between 10 and 14 weeks after the end of spa treatment period by VAS, WOMAC, and the SF-36 Health Survey [32, 33, 37].

The study in Denizli SPA (Turkey) evaluated knee OA patients treated with mud-pack therapy rich in organic substances (lignin and humin) for 30 minutes/day for 15 days in 3 weeks. Patients were required to suspend any analgesic therapy during trial except in case of intense pain. The control group patients continued ongoing pharmacological treatment [30]. The results were evaluated at the baseline, after the end of spa therapy, and 30 days after the end of the treatment by VAS, WOMAC, and the patient's and physician's global assessments of disease status and response to therapy scores [32, 33].

The study performed in three* French SPAs (Aix-les-Bains, Balaruc*,* and Dax)* on 382 patients tested the effects of 18 spa therapy (massages, showers, mud, and pool sessions) cycles over three weeks (features of mineral water not indicated). Ongoing therapy was allowed in experimental and control groups. Follow-up was at 1, 3, and 6 months after the beginning of the treatment [25]. The patient's and physician's opinion and the Minimal Clinic Important Improvement (MCII) were adopted for the measurement of results [38].

## 3. Results

The main results reported by the authors of each study are summarized below. In the study by Fraioli et al., the parameters used to measure the severity of the symptoms of knee OA (VAS, Lequesne's Index, flexion/extension, and tenderness) were significantly reduced after 3 mud-bath therapy cycles during 1 year; in the control group no significant differences were observed. Even in the comparison between the patients of the two groups that were following the therapy with drug, the results were that, in experimental group, the percentage of patients with no symptoms or mild symptoms was higher than that in control group [20]. In the studies, Fioravanti et al. observed a significant improvement of all evaluated parameters VAS, Lequesne's Index, W-TPS (WOMAC Total Pain Score), W-TSS (WOMAC Total Stiffness Score), and W-TFS (WOMAC Total Function Score) at the end of the cycle of mud-bath therapy, whereas in the control group no significant differences were noted [21–23]. Fioravanti et al. found that adiponectin and resistin were reduced after mud-bath therapy in patients with knee OA, while no decrease was detected in the control group [21, 23].

Cantarini et al. evaluated results by VAS and Lequesne's Index and observed a marked improvement in the first experimental group (mud-bath therapy) at both 3 and 12 weeks of treatment, an improvement at 3 weeks in the second experimental group (short wave diathermy on both knees) and a deterioration at both steps in the control group. Also AIMS was improved in others steps. All the groups continued ongoing therapy. The drugs dosage in the groups varied according to pain intensity: in the first experimental group a significant reduction in drug intake was observed at both steps versus baseline (before starting treatment), in the second experimental group the reduction was observed at 3 weeks versus baseline time, and in the control group an increase in drugs intake was observed at both 3 and 12 weeks in comparison with baseline [24].

Kovacs et al. reported a significant amelioration of initial pain, tenderness on palpation, walking ability, time to climb and descend 20 steps, and patient's and physician's opinion in patients treated with bath therapy and three months later this improvement persisted only in the actively treated group. The values reduction were significant for experimental group and not significant for control group [26].

Moreover, Tishler et al. found that balneotherapy was effective in improving all measuring indexes (VAS, WOMAC, and Lequesne's Index), except for WOMAC-Stiffness Index, as well as a reduction in analgesic and NSAID consumption, which was also noted in experimental group. The values reduction were significant for experimental group and not significant for control group, and the improvement remained significant after 10 weeks in the experimental group [31].

Karagülle et al. by using Lequesne Algo Functional Index (LAFI) and VAS, as well as the time taken to climb and descend 10 steps, to make a 15-foot walk and to crouch and raise three times observed a significant reduction in LAFI and VAS which was found at week 2, week 12, and week 24 in the bath therapy group compared to baseline. Comparing the two groups (experimental and control) differences, bath therapy was superior to drug therapy in pain reduction and in physician's global assessment at all time points [29].

Kulish et al. evaluated results by VAS, WOMAC, right and left knee bend angle measured with goniometer, circumference of the right and left knee, time to climb 22 stairs of the Stage Climb Time (SCT), and patient questionnaire on quality of life in relation to health (Quality of life) observed before, immediately after treatment, and after 15 weeks. The obtained results were more favourable in the experimental group than in the control group with regard to the various parameters considered, even after 15 weeks, except for those referring to the knee circumferences and time used for climb the 22 steps [27].

In the study, Gaal et al. compared baselines and all monitored parameters (VAS, WOMAC, and SF-36) were significantly improved by balneotherapy after 2 and 14 weeks of therapy. Moreover, patients taking NSAIDs dropped from 60.5% to 10.5% and 0% for 2 and 14 weeks of SPA treatment [28].

Odabaşi et al. used VAS and WOMAC scales for the evaluation of results as well as a global health status expressed by the patient's and the physician's assessment of disease status. A significant decrease was observed in both groups (superior in the study group as compared to the controls) in terms of disease severity index scores. Compared to the baseline an improvement was observed in the experimental group in all the considered parameters 3 weeks after the beginning of the study, and a greater improvement was observed 7 weeks after the beginning of the study. The number of patients who had MCII was significantly higher in the study group at week 3 and remained high till the end of the follow-up period [30].

Finally Forestier et al. reported that a three-week period of spa treatment combined with a pharmacologic and home exercise program was superior to conventional treatments and exercise alone at the end of sixth month, and it was better tolerated. The author found that the percentage of patients who reached the MCII was significantly higher in the experimental group than in the control group, 50,8% versus 36,4%, and a significant reduction of VAS and WOMAC was observed after 6 months from the end of treatment. The overall opinions of the patient (SF36 scores) and the examining physician at 6 months showed an improvement in both groups (54.4%) [25].

## 4. Discussion

Therapy of knee osteoarthritis is based on pharmacological and nonpharmacological measures. These include the use of SPA therapy (comprises a broad spectrum of therapeutic modalities including hydrotherapy, balneotherapy, mud-pack therapy, mud-bath therapy, massage, and exercise) to supplement or alternate pharmacological and/or physiotherapeutic therapy, with favourable results on pain, drug use, and improving the patient's general well-being. The biological effects of mud-bath therapy in osteoarthritis are mainly secondary to thermal and chemical stimuli. The thermal effects are characterized by an increase in the temperature of the skin, subcutaneous tissue, and muscles, with decreasing muscular tone. Hyperaemia at periarticular sites (capsules, ligaments, and tendon insertions) caused by heat stimulation of the thermal mud can contribute to the removal of inflammatory cytokines and chemokines thus reducing pain [19, 39]. The heat component plays a fundamental role together with the organic and inorganic properties of the thermal medium (mineral content) [40, 41]. Numerous studies have highlighted the effects of mud-pack therapy, bath therapy, or mud-bath therapy on prostaglandin E2 (PGE2), leukotriene B4 (LTB4), tumor necrosis factor-alpha (TNF-*α*), interleukin-1*β* (IL-1*β*), interleukin-6 (IL-6), metalloproteinases (MMP-3), prolactin and growth hormone (GH), insulin-like growth factor I (IGF1), transforming growth factor beta (TGF-*β*), reactive oxygen species (ROS), catalase, malondialdehyde (MDA), superoxide dismutase (SOD) and glutathione peroxidase (GSH-peroxidase), nitric oxide (NO) and myeloperoxidase (MPO), adiponectin, cartilage oligomeric matrix protein (sCOMP), and systemic modification of the chondrocyte markers, suggesting a protective action on articular cartilage [42–48]. The general effect of mud-bath therapy with thermal waters is achieved through influences on the hypothalamus-pituitary-adrenal axis, with increased secretion of Adreno Cortico Tropic Hormone (ACTH) and cortisol and greater production of endogenous opioids (*β*-endorphin) [49, 50].

Some systematic reviews on spa therapy for knee OA have recently been published [51–55].

Because the studies we reviewed differed markedly from one another in terms of the methods used, we were unable to conduct a quantitative analysis (meta-analysis) of pooled data from the 12 studies. We finally summarized the results obtained from the literature on the basis of the main outcomes of the different studies included in this review. The results of SPA therapy according to measures employed in the studies evaluated are shown in Table 5.

Concerning the VAS, 297 patients were homogeneously evaluated among 12 studies. The mean VAS values before and after SPA therapy were 54.71 and 29.36, respectively, showing a significant reduction corresponding to 46.34% if compared with the initial value (Table 5-line 1).

With regard to Lequesne's Index, it was used in 6 of the studies included in this review over a total of 140 patients in the SPA treated groups [20, 22–24, 29, 31]. Mean Lequesne's Index significantly improved with an overall improvement of 33.78% compared to the initial value (Table 5-Line 2).

The WOMAC was evaluated in 7 studies. WOMAC includes three different measurement scales: WOMAC Total Pain Score (TPS), WOMAC Total Stiffness Score (TSS), and WOMAC Total Physical Function Score (TPFS). Four studies [21, 22, 27, 31] evaluated results from all the three WOMAC scales; in other two researches [25, 28] only the third version (W-TPFS) was measured; finally, in the last study [30] a global index was derived from the sum of scores of the three WOMAC scales. WOMAC Total Pain Score was examined in 4 studies, for a total of 170 patients [21, 22, 27, 31]. WOMAC improved before and after SPA therapy from 21.49 to 13.74, respectively, suggesting a decrease in pain of 36.06% (considering the initial value = 100) (Table 5-line 3).

Similarly, the results evaluated with the WOMAC Total Stiffness Score (total patients =126), were equally favourable. In fact, the mean values of the index calculated on the total of patients before and after the treatment were 27.78 and 16.98, respectively. Hence, WOMAC was reduced by 38.87% (Table 5-line 4). Finally, the third WOMAC scale (WOMAC Total Physical Function Score) was investigated in 6 studies [21, 22, 25, 27, 28, 31]. The total number of patients for the evaluation was 387; the mean scores were 40.26 and 26.71 before and after therapy, respectively. Thus, reduction was 33.65% from the initial WOMAC value (Table 5-line 5).

AIMS was evaluated in 2 studies [22, 24] and SF36 in other 2 studies [25, 28]. AIMS was improved in all studies suggesting that psychological aspects, such as anxiety and depression, can also be positively influenced by SPA therapy. SF36 also improved in all considered studies. Two studies evaluated adipokines, specifically adiponectin, resistin, and visfatin, showing that adiponectin and resistin can be reduced by mud-bath therapy [21, 23].

## 5. Conclusions

To conclude, SPA therapy on the ground of the researches objective of this review is effective in the management of knee OA and significantly improves the pain and functional status of patients with knee OA. SPA therapy is a noninvasive, complication-free, and cost-effective alternative modality for the conservative treatment of knee osteoarthritis.

The mud-pack therapy, bath therapy, mud-bath therapy, and SPA therapy have proved to be effective in the treatment and in preventing progression of the disease and the onset of disability (secondary prevention) of knee osteoarthritis, by reducing pain and functional limitation and improving quality of life of affected patients. Turkish League against Rheumatism states that balneotherapy may be recommended for at least two weeks of treatment because of its thermal and nonthermal effects. In addition to this treatment, peloidotherapy may be advised [56]. In the OARSI guidelines for the nonsurgical management of knee osteoarthritis, balneotherapy was considered appropriate only for the subphenotype with multiple-joint OA (symptomatic OA of the knee) in addition to other joints (e.g., hip, hand, and spine) and comorbidities (diabetes, hypertension, cardiovascular disease, renal failure, gastrointestinal bleeding, depression, physical impairment limiting activity, and obesity) due to paucity of treatment alternatives for that group [57]. Indications based on the clinical guidelines of the French National Authority suggest that patients with knee osteoarthritis might gain the benefit of a persistent improvement (at least twelve weeks) of pain, analgesic and nonsteroidal anti-inflammatory drug consumption, functional capacity, and/or quality of life [58].

RCTs findings suggest that spa therapy is part of an integrated and synergistic multidisciplinary approach with other treatments (pharmacotherapy, physiotherapy), allowing also the reduction of conventional treatment dosage, possibly resulting in lesser costs and drug-related adverse events, and further improving the quality of life of affected patients. Moreover, patients benefit from staying in an environment with a favorable and relaxing climate with a positive impact on their perception of wellbeing [59].

## Figures and Tables

**Table 1:** Mud-bath therapy: number of patients included in the experimental and control groups.

Study	Treated Patients	Treatment	Control Group	Ongoing treatment	Total Patients
Fraioli A. et al. 2011	17	Mud-bath therapy	44	yes	61
Fioravanti A. et al. 2010	40	Mud-bath therapy	40	yes	80
Fioravanti A.et al. 2011	30	Mud-bath therapy	-* *-	-* *-* *-	30
Fioravanti A. et al. 2015	49	Mud-bath therapy	46	yes	95
Cantarini et al. 2007	30+24 (°)	Mud-bath therapy (*∗*)	20	yes (°°)	74
Total Patients	190		150		340

(°) 2 experimental groups included, the first one treated by mud-bath therapy, the second one with physical therapy.

(*∗*) Ongoing treatment.

(°) Not allowed any physical or pharmacological treatment during the study.

(°°) Not allowed glucocorticoids and intra-articular treatment with hyaluronic acid.

**Table 2:** Bath therapy: number of patients included in the experimental and control groups.

Study	Treated Patients	Treatment	Control Group	Ongoing treatment	Total Patients
Gaal J et al. 2008	38	Bath therapy (#)	-* *-* *-	-* *-* *-	38
Kovàcs I. et al. 2002	31	Bath therapy (°)	27	yes	58
Tishler M., et al. 2004	48 (§)	Bath therapy (#)	24	yes	72
Karagülle M. et al. 2007	10 (^∧^)	Bath therapy (#)	10	yes	20
Kulisch A et al. 2014	38	Bath therapy (##)	39	yes	77
Total Patients	165		100		265

(§) 4 patients did not complete the study.

(^∧^) 1 patient has not completed the study.

(°) 2 experimental groups included, the first one treated by balneotherapy, the second one with physical therapy.

(*∗*) Ongoing treatment.

(#) Allowed NSAIDs treatment.

(°) Not allowed any physical or pharmacological treatment during the study.

(##) Not allowed physical treatment and changes in ongoing NSAIDs therapy.

**Table 3:** Mud-pack therapy: number of patients included in the experimental and control group.

Study	Treated Patients	Treatment	Control Group	Ongoing treatment	Total Patients
Odabaşi E et al. 2009	32	Mud-pack therapy (*∗∗*)	25	yes	57

(*∗∗*) Allowed treatment with analgesic drugs in case of worsening pain, by informing the study authors.

**Table 4:** Spa therapy: number of patients included in the experimental and control group.

Study	Treated Patients	Treatment	Control Group	Ongoing Treatment	Total Patients
Forestier R.et al. 2010	195	Multiple interventions in spa resorts	187	yes	382

**Table 5:** Results of SPA therapy according to measures employed in the studies evaluated.

Measure scales applied	Number of patients (1)	Mean values		b1- b2	Reduction % (2)
		b1 before treatment	b2 after treatment		
VAS	297	54.71	29.36	- 25.35	- 46.34
Lequesne's Index	140	56.61	37.49	- 19.12	- 33.78
WOMAC – TPS (3)	170	21.49	13.74	- 7.75	- 36.06
WOMAC – TSS (4)	126	27.78	16.98	- 10.80	- 38.87
WOMAC – TPFS (5)	387	40.26	26.71	- 13.55	- 33.65

(1) Number of patients included in the experimental groups participating in the studies using a specific measure scale, excluding the studies in which data are expressed differently from practice (for instance, with percentage but without absolute values, only with a graphic representation, etc.).

(2) Calculated according to the following procedure: values at the treatment end if the starting values are = 100 (referring to VAS) 29,36:54,71 = x:100; x = 53,66 = value observed at the end of treatment if starting value is 100; 100-53,66 = 46,34 = percentage reduction of VAS value at the treatment end.

(3) TPS =Total Pain Score.

(4) TSS =Total Stiffness Score.

(5) TPFS =Total Physical Function Score.
